# m6A-TSHub: Unveiling the Context-specific m^6^A Methylation and m^6^A-affecting Mutations in 23 Human Tissues

**DOI:** 10.1016/j.gpb.2022.09.001

**Published:** 2022-09-09

**Authors:** Bowen Song, Daiyun Huang, Yuxin Zhang, Zhen Wei, Jionglong Su, João Pedro de Magalhães, Daniel J. Rigden, Jia Meng, Kunqi Chen

**Affiliations:** 1 Key Laboratory of Gastrointestinal Cancer (Fujian Medical University), Ministry of Education, School of Basic Medical Sciences, Fujian Medical University, Fuzhou 350004, China; 2 Department of Mathematical Sciences, Xi’an Jiaotong-Liverpool University, Suzhou 215123, China; 3 Institute of Systems, Molecular and Integrative Biology, University of Liverpool, Liverpool L69 7ZB, United Kingdom; 4 Department of Biological Sciences, Xi’an Jiaotong-Liverpool University, Suzhou 215123, China; 5 Department of Computer Science, University of Liverpool, Liverpool L69 7ZB, United Kingdom; 6 Institute of Ageing & Chronic Disease, University of Liverpool, Liverpool L69 7ZB, United Kingdom; 7 School of AI and Advanced Computing, Xi’an Jiaotong-Liverpool University, Suzhou 215123, China; 8 AI University Research Centre, Xi’an Jiaotong-Liverpool University, Suzhou 215123, China

**Keywords:** *N*^6^-methyladenosine, Context-specific analysis, Cancer mutation, Genome analysis, Functional annotation

## Abstract

As the most pervasive epigenetic marker present on mRNAs and long non-coding RNAs (lncRNAs), ***N*^6^-methyladenosine** (m^6^A) RNA methylation has been shown to participate in essential biological processes. Recent studies have revealed the distinct patterns of m^6^A methylome across human tissues, and a major challenge remains in elucidating the tissue-specific presence and circuitry of m^6^A methylation. We present here a comprehensive online platform, m6A-TSHub, for unveiling the context-specific m^6^A methylation and genetic mutations that potentially regulate m^6^A epigenetic mark. m6A-TSHub consists of four core components, including (1) m6A-TSDB, a comprehensive database of 184,554 functionally annotated m^6^A sites derived from 23 human tissues and 499,369 m^6^A sites from 25 tumor conditions, respectively; (2) m6A-TSFinder, a web server for high-accuracy prediction of m^6^A methylation sites within a specific tissue from RNA sequences, which was constructed using multi-instance deep neural networks with gated attention; (3) m6A-TSVar, a web server for assessing the impact of genetic variants on tissue-specific m^6^A RNA modifications; and (4) m6A-CAVar, a database of 587,983 The Cancer Genome Atlas (TCGA) **cancer mutations** (derived from 27 cancer types) that were predicted to affect m^6^A modifications in the primary tissue of cancers. The database should make a useful resource for studying the m^6^A methylome and the genetic factors of epitranscriptome disturbance in a specific tissue (or cancer type). m6A-TSHub is accessible at www.xjtlu.edu.cn/biologicalsciences/m6ats.

## Introduction

Among the more than 150 distinct chemical modifications naturally decorating cellular RNAs [1], *N*^6^-methyladenosine (m^6^A) is the most pervasive marker on mRNAs and long non-coding RNAs (lncRNAs), and has been associated with a number of essential biological functions and processes [2], [3], including mRNA stability [4], splicing [5], translation [6], [7], heat shock [8], DNA damage [9], and embryonic development [10]. Increasing evidence has indicated a critical role of m^6^A dysregulation in various human diseases, especially multiple cancers, such as breast cancer [11], [12] and prostate cancer [13]. For example, inhibition of an m^6^A methyltransferase (METTL13) could be used as a potential therapeutic strategy against acute myeloid leukemia [14].

Developed in 2012, m^6^A-seq (methylated RNA immunoprecipitation sequencing; MeRIP-seq) was the first whole transcriptome m^6^A profiling approach [15], [16]. It relies on antibody-based enrichment of the m^6^A signals, enabling the identification of m^6^A-containing regions with a resolution of around 100 nt. Currently, m^6^A-seq is still the most popular m^6^A profiling approach and has been applied in more than 30 different organisms. Besides m^6^A-seq, recent advances in integration of ultraviolet cross-linking, enzymatic activity, and domain fusion have offered improved even base-resolution m^6^A detection through techniques such as, miCLIP/m^6^A-CLIP-seq [17], [18], m^6^A-REF-seq [19], and DART-seq [20]. However, compared with m^6^A-seq, these approaches require more complicated experimental procedures and have therefore been applied in fewer biological contexts.

To date, more than 120 computational approaches have been developed for the computational identification of RNA modifications [21], [22] from the primary RNA sequences. These include the iRNA toolkits [23], [24], [25], [26], [27], [28], [29], [30], [31], MultiRM [32], DeepPromise [22], RNAm5CPred [33], SRAMP [11], Gene2vec [34], PEA [35], PPUS [36], WHISTLE [37], m5UPred [38], WeakRM frameworks [39], [40], m6ABoost [41], PULSE [42], m6AmPred [43], BERMP [44], and MASS [45]. Together, these efforts have greatly advanced our understanding of multiple RNA modifications in different RNA regions and in various species (see recent reviews [22], [46], [47], [48]). A number of epitranscriptome databases have been constructed. MODOMICS collects the pathways related to more than 150 different RNA modifications [1]. RMBase [49], m^5^C-Atlas [50], and m^6^A-Atlas [51] assembled millions of experimentally validated m^6^A and m^5^C sites. REPIC has been established as a comprehensive atlas for exploring the association between m^6^A RNA methylation and chromatin modifications [52]. ConsRM provides the conservation score of individual m^6^A sites at the base resolution, which can be used to differentiate the functionally important and ‘passenger’ m^6^A sites [53]. m6A2Target compiles the target molecules of m^6^A methyltransferases, demethylases, and binding proteins [54]. This work has extended our knowledge of the functional epitranscriptome, and greatly facilitated relevant research. Special efforts have also been made to explore the effects of genetic variants on RNA modifications and their association with various diseases. m6AVar [55] was the first database that focused on the genetic factors related to epitranscriptome disturbance. It documented more than 400,000 m^6^A-affecting genetic variants, which were further labeled with disease and phenotype associations identified from genome-wide association study (GWAS) analysis. This prediction framework was improved and later applied to eight other RNA modifications (m^5^C, m^1^A, m^5^U, Ψ, m^6^Am, m^7^G, and 2′-O-Me, and A-to-I) by RMVar [56] and RMDisease [57]. These aforementioned databases systematically revealed the general association between epitranscriptome layer dysregulation and various diseases (see a recent review [58]).

Existing computational approaches for epitranscriptome analysis have been quite successful in providing a large quantity of useful information; however, most of them failed to consider the tissue specificity of m^6^A epi-transcriptome [59], [60]. Indeed, a recent study by Liu et al*.* unveiled distinct tissue-specific signatures of the m^6^A epitranscriptome in human and mouse [61], which are induced by context-specific expression of m^6^A regulators [methyltransferases, demethylases, and RNA-binding proteins (RBPs)] [62] and genetic drivers [63]. Nevertheless, most existing approaches for RNA modification site prediction completely ignore the context specificity of the epitranscriptome and simply assume a single model for different tissues, undermining their accuracy and applicability. To the best of our knowledge, the only three approaches that clearly support the identification of tissue-specific m^6^A methylation are im6A-TS-CNN [64], iRNA-m6A [65], and TS-m6A-DL [66], all covering only three human tissue types (brain, liver, and heart). Similarly, when screening for the genetic variants that can affect RNA modifications, previous work assumes a consistent influence in different tissues (see Table S1 for a detailed description and comparison). However, because different epitranscriptome patterns were observed among different tissues, genetic mutations that can alter m^6^A methylation in one tissue may not necessarily function similarly in a different tissue. Likewise, there are significant differences in incidence, mortality, and molecular signatures across cancer originating from different tissues [67], [68]. It is therefore highly desirable to develop approaches that could take full advantage of the tissue-specific RNA methylation profiles so as to make more reliable predictions with respect to a specific tissue type [69]. This is particularly critical for studying the epitranscriptome circuits of diseases that are explicitly associated with a specific tissue, such as, cancers.

To address this issue, we present here a comprehensive online platform m6A-TSHub for unveiling the context-specific m^6^A methylation and m^6^A-affecting mutations in 23 human tissues. m6A-TSHub consists of four core components: (1) m6A-TSDB, a database for 184,554 experimentally validated m^6^A-containing peaks (m^6^A sites) derived from 23 distinct human normal tissues and 499,369 m^6^A-containing peaks (m^6^A sites) from 25 matched tumor conditions, extracted from 233 m^6^A-seq samples, respectively; (2) m6A-TSFinder, an integrated online server for the prediction of tissue-specific m^6^A modifications in 23 human tissues, built upon a gated attention-based multi-instance deep neural network; (3) m6A-TSVar, a web server for systemically assessing the tissue-specific impact of genetic variants on m^6^A RNA modification in 23 human tissues; (4) m6A-CAVar, a database of 587,983 The Cancer Genome Atlas (TCGA) cancer mutations (derived from 27 cancer types) that may lead to the gain or loss of m^6^A sites in the corresponding cancer-originating tissues.

**Figure 1:**
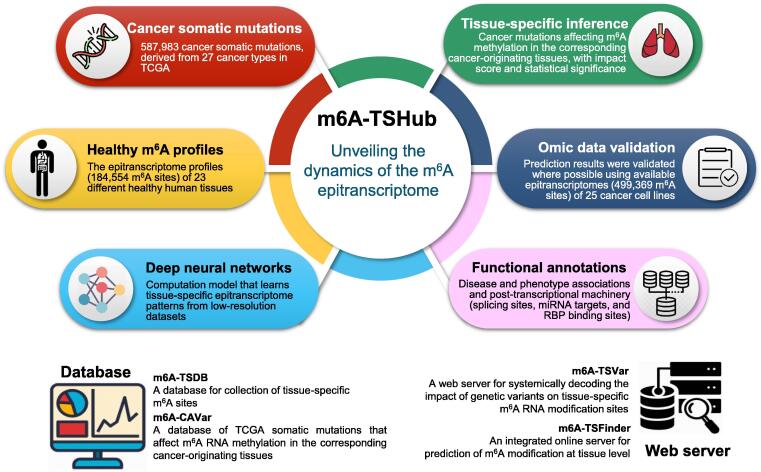
**The overall design of m6A-TSHub** By integrating 184,554 m^6^A sites detected from 23 different healthy human tissues (m6A-TSDB), a deep learning framework that learns tissue-specific RNA methylation patterns was developed (m6A-TSFinder). The effect of genetic variants on tissue-specific m^6^A sites was then evaluated (m6A-TSVar). A total of 587,983 cancer somatic mutations were predicted to be able to affect m^6^A methylation of RNA in their corresponding cancer-originating tissues. The predicted m^6^A-affecting SNPs were then systematically validated using available cancer epitranscriptome datasets, and then functionally annotated with disease and phenotype associations from GWAS, along with features relating to the post-transcriptional machinery (miRNA target sites, splicing sites, and RBP binding sites) that are potentially mediated by m^6^A modification (m6A-CAVar). A web interface was constructed to enable the exploration, query, online analysis, and download of relevant results and data. m^6^A, *N*^6^-methyladenosine; TCGA, The Cancer Genome Atlas; GWAS, genome-wide association study; RBP, RNA-binding protein; miRNA, microRNA.

In addition, the m^6^A-associated variants were also annotated with their potential post-transcriptional regulatory roles, including RBP binding regions, microRNA (miRNA) targets, and splicing sites, along with their known disease and phenotype linkage integrated from GWAS catalog [70] and ClinVar databases [71]. The m6A-TSHub is freely accessible at www.xjtlu.edu.cn/biologicalsciences/m6ats, and should be a useful resource for studying the m^6^A methylome and genetic basis of epitranscriptome disturbance with respect to a specific cancer type or tissue. The overall design of m6A-TSHub is shown in Figure 1.

## Data collection and processing

### Data resource — m6A-TSDB

We collected the epitranscriptome profiles of 23 healthy human tissues, from which the tissue-specific RNA methylation patterns were learned using deep neural networks. Specifically, the raw sequencing data of 78 m^6^A-seq samples were downloaded directly from Gene Expression Omnibus (GEO) repository of National Center for Biotechnology Information (NCBI) [72] and National Genomics Data Center (NGDC) [73] (Table S2). Adaptors and low-quality nucleotides were removed by Trim Galore [74], followed by quality control using FastQC. The processed reads were then aligned to the reference genome GRCh37/hg19 by HISAT2 [75]. The m^6^A-enriched regions (peaks) located on transcripts were detected by exomePeak2 [76] using its default setting with GC contents corrected. In total, m^6^A profiling samples from 23 healthy human tissues (184,554 m^6^A-containing peaks) were processed. We filtered all obtained m^6^A-enriched regions to retain peaks with at least one DRACH consensus motif and used these peak regions containing tissue-specific m^6^A signals as positive data. Negative data were randomly collected from non-peak regions located on the same transcript of the corresponding positive data, and cropped to balance the length and number between positive and negative regions (with a positive-to-negative ratio of 1:1). The genomic sequences of both positive and negative regions were then extracted for developing the tissue-specific m^6^A prediction model.

To evaluate the effect of cancer somatic variants on m^6^A methylation in their originating tissues, a total of 2,587,191 cancer somatic variants from 27 different cancer types were obtained from TCGA (release version v27.0-fix) [77] (Table S3). Meanwhile, 155 m^6^A-seq samples profiling the epitranscriptome (499,369 m^6^A-containing peaks) of 25 cancer cell lines (corresponding to 17 tissue types) were also obtained using the same data processing pipeline (Table S2), which were used for the validation of the predicted effects on m^6^A methylation of the variants (detailed in the following).

### Learning tissue-specific m^6^A methylation with deep neural networks **—** m6A-TSFinder

**Figure 2:**
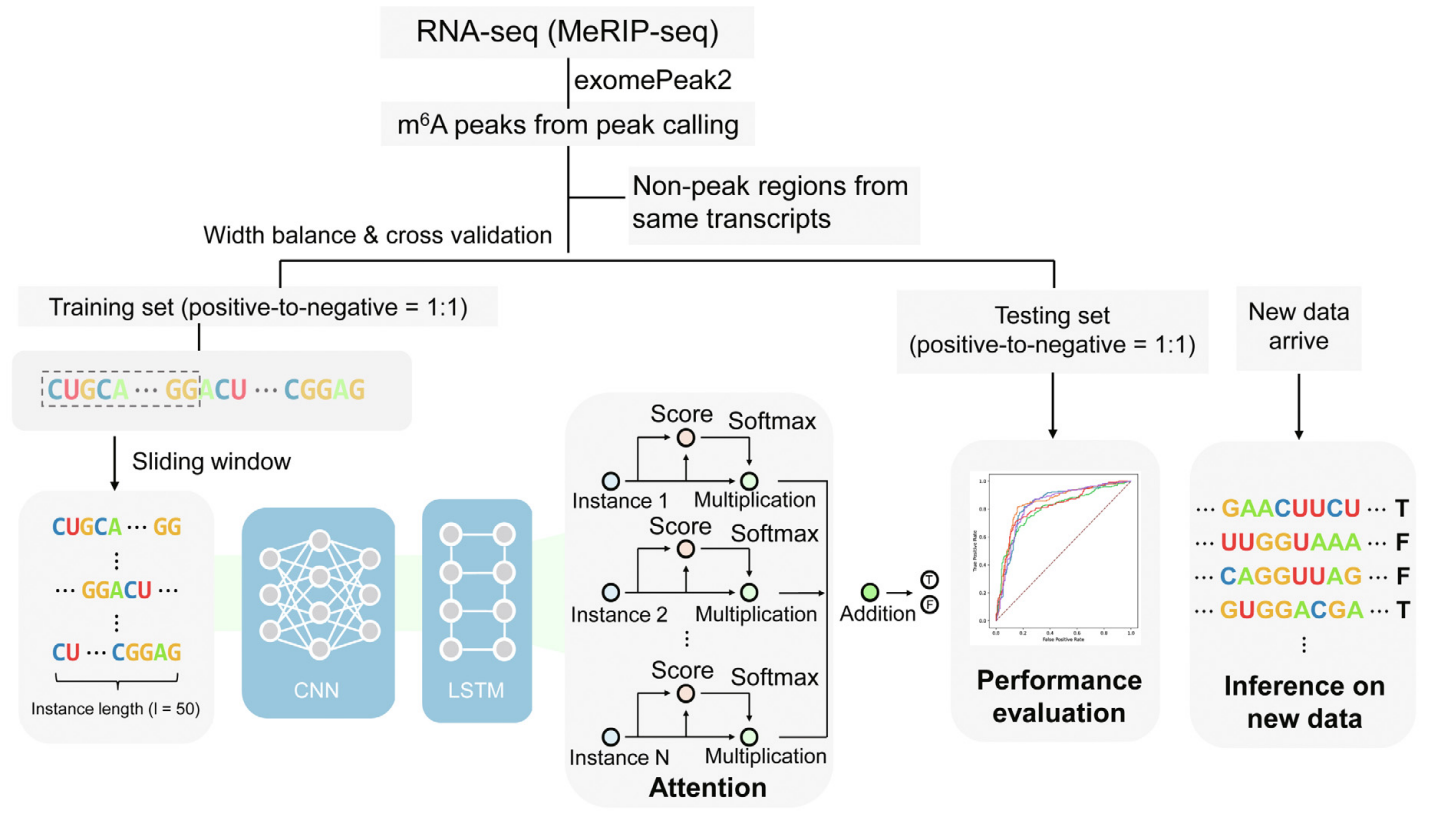
**A simplified graphic illustration of the proposed m6A-TSFinder framework** RNA-seq, RNA sequencing; MeRIP-seq, methylated RNA immunoprecipitation sequencing; CNN, convolutional neural network; LSTM, long short-term memory.

The purpose of weakly supervised learning is to develop predictive models by learning from weakly labeled data, such as m^6^A peaks of low resolution detected by the m^6^A-seq (or MeRIP-seq) technique [15], [16]. Unlike supervised learning based on single-nucleotide resolution data, it works for the case in which only coarse-grained labels (indicating whether a genome bin contains an m^6^A site) are available for these peaks of various lengths. We previously proposed a general weakly supervised learning framework WeakRM [78], which takes labels at the sequence level (rather than a nucleotide level) as input and predicts the sub-regions that are most likely to contain the RNA modification. As a simplified illustration shown in Figure 2, the m6A-TSFinder framework is divided into several sub-sections. Firstly, multi-instance learning treats each entire RNA sequence as a ‘bag’, with multiple ‘instances’ within the ‘bag’ determined by a fixed-length sliding window. Previous studies have shown that a 40–50-nt context region is sufficient for modification predictions. Therefore, in m6A-TSFinder, a sliding window of 50 nt was used, which was also helpful in improving the prediction resolution. Secondly, the RNA instances were fed into the m6A-TSFinder model using one-hot encoding, which is widely used in deep learning-based models. The extracted instances pass through the same feature extraction module (the weights of the network are shared in this module) and output instance-level features. The network architecture of the feature extraction section used in m6A-TSFinder includes the first convolutional layer to capture motifs, a max-pooling layer to remove weak features and enlarge the receptive field, a dropout layer that prevents overfitting in training, and a second convolutional layer which learns local dependencies among motifs. In order to further improve the performance of the model, in m6A-TSFinder, we use a long short-term memory (LSTM) layer to replace the second convolutional layer, so that the model can learn the long-range dependence of the motif while maintaining local dependence. Lastly, gated attention was used as the score function to obtain bag-level probabilities from multiple instance-level features. The gated attention module consists of three fully connected layers. The first two layers learn hidden representations from the instance features using tanh and sigmoid activation functions. Their element-wise multiplication is then sent to the third fully connected layer, which learns the similarity between the product and a context feature vector and outputs an attention score for each instance. The score is further normalized using the softmax function, so that the weights of all instances add up to 1. The weighted summation of instance features is treated as the bag-level feature and used to output the final probability score. Together, our model can be trained end-to-end using the binary cross-entropy loss calculated by the bag-level label. Our model was trained using the Adam optimizer under the Tensorflow framework. The learning rate was initially set to 1E−4, and gradually decayed to 1E−5 during the training process of 20 epochs. It is worth mentioning that when the number of instances is consistently set to 1, the weight of the instance is always 1, and the label becomes the instance level. In that case, the gated attention module is degraded, and the network becomes a strong supervised learning framework with two feature extraction layers.

### Decoding the tissue-specific effect of variants on m^6^A methylation — m6A-TSVar & m6A-CAVar

**Figure 3:**
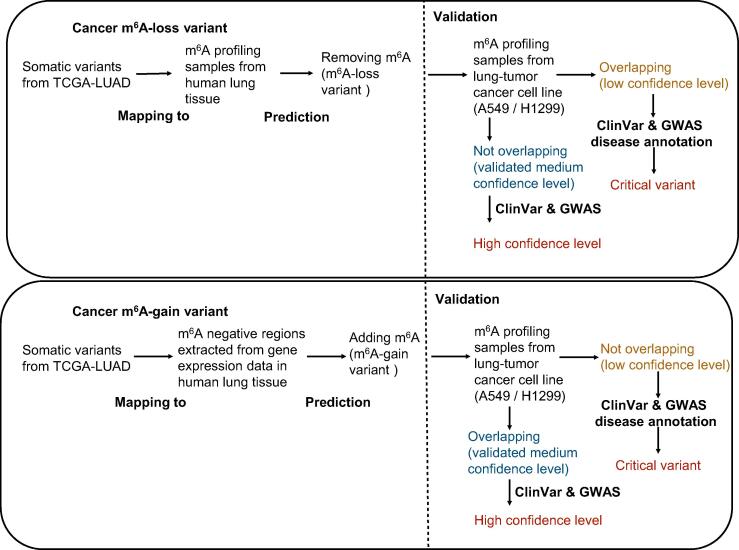
**Workflow of how to determine the confidence level of m^6^A variants** Three types of confidence levels were applied. The cancer-driving somatic variants were extracted from TCGA projects, and mapped to the m^6^A profiling samples derived from corresponding tumor-growth tissues. A tissue-specific weakly supervised model was then applied to obtain m^6^A-associated variants classified into the low-confidence level group. m^6^A profiling samples from tumor-growth tissues were then used for validation of the prediction results, and the validated portion was classified into the medium-confidence level group. Lastly, all variants with a medium confidence level were annotated with disease information from ClinVar and GWAS, and then classified into the high-confidence level group. Lung tissue, healthy and cancerous, is used as an example here. The same protocol was followed for all 23 tissues. GWAS, genome-wide association study.

Similar to previous studies [55], [56], [79], [80], a cancer somatic variant is defined as a tissue-specific m^6^A variant if it could lead to the gain or loss of m^6^A methylation in a specific tissue. The tissue-specific inference was made possible by our deep neural network model m6A-TSFinder. Specifically, the predicted tissue-specific m^6^A variants were further classified into three confidence levels — low, medium, and high (Figure 3).

#### Low confidence level

An m^6^A-associated variant with a low confidence level was defined directly by the tissue-specific prediction model. For example, a synonymous somatic variant (Chr5:92929473, positive strand, C > T, TCGA barcode: TCGA-49-6742-01A-11D-1855-08) was extracted from The Cancer Genome Atlas Lung Adenocarcinoma (TCGA-LUAD) project, which was then predicted to eliminate the methylation of an experimentally validated m^6^A-containing region (Chr5:92929314-92929786, positive strand) originally detected in human lung tissue [61].

#### Medium confidence level

The m^6^A variants with a medium confidence level are those that can be verified on available epitranscriptome data from cancer samples originating from the matched tissue. After the low confidence level mentioned above, by checking the m^6^A-containing regions reported in lung adenocarcinoma cancer cell lines A549 [81] and H1299 [82], we confirmed that no m^6^A peaks were further observed in A549 and H1299 for the variant-affected region (Chr5:112176059-112176334, positive strand). Consequently, this LUAD somatic variant was upgraded to a ‘medium’ confidence level in the m6A-CAVar database. It is worth noting that the predicted m^6^A dynamics in m6A-CAVar were systematically validated using available epitranscriptome datasets from the matched healthy and cancer samples, providing another layer of quality assurance from real omic datasets: existing approaches only use those datasets to provide the m^6^A site information without searching for potential evidence of m^6^A status switching.

#### High confidence level

Only a very small number of variants have been clearly associated with diseases and phenotypes unveiled by GWAS analysis, and are known as disease-TagSNPs. These variants exhibited their clinical significance and are very likely to be functionally important. Thus, m^6^A variants of ‘high’ confidence level were defined as the validated m^6^A variants that can also be mapped to disease-TagSNPs extracted from ClinVar [71] and GWAS catalog [70], while those not validated were referred to as ‘critical’.

Additionally, the association level (AL) between an SNP and m^6^A RNA modification was defined as follows:

(1) $$\text{AL}=\left\{\begin{array}{cc} \text{2}P_{\mathrm{SNP}}-2\max\left(0.5,P_{\mathrm{WT}}\right) & \text{for gain}\\ 2P_{\mathrm{WT}}-2\max\left(0.5,P_{\mathrm{SNP}}\right) & \text{for loss} \end{array}\right)$$

where $P_{\mathrm{WT}}$ and $P_{\mathrm{SNP}}$ represent the probability of m^6^A RNA modification for the wild-type and mutated sequences, respectively. The AL ranges from 0 to 1, with 1 indicating the maximum impact on m^6^A methylation. The statistical significance was assessed by comparing the ALs of all mutations, with which the upper bound of the *P* value can be calculated from its absolute ranking. The m^6^A-associated variants with AL > 0.4 and *P* < 0.1 were retained. We also considered the possibility of a variant destroying a part of (but not an entire) m^6^A peaks. For peaks wider than 500 nt, the impacts were also evaluated on the 200-nt flanking regions of the variant.

The predicted m^6^A variants were then validated on the epitranscriptome datasets from the matched healthy and cancer samples. We consider a prediction validated by omic data if the matched dynamics of m^6^A sites were observed under the healthy tissue and the cancer samples with the same tissue origin. It may be worth noting that, omic data were only used to inform the prediction of m^6^A sites in previous studies [55], [56], [79], [80]; however, our analysis also relies on it to confirm the predicted disturbance of m^6^A status between the healthy and cancer conditions. This extra layer of confirmation directly from available omic datasets should effectively enhance the reliability of our database.

### Functional annotation

The identified m^6^A variants were annotated with various information, including transcript region [coding sequence, 3′ untranslated region (UTR), 5′ UTR, start codon, and stop codon], gene annotation (gene symbol, gene type, and Ensembl gene ID), evolutionary conservation (phastCons 60-way), deleterious level by SIFT [83], PolyPhen2 HVAR [84], PolyPhen2HDIV [84], LRT [85] and FATHMM [86] using the ANNOVAR package [87], absolute ranking by comparing with the ALs of all mutations (top 1% and top 5%), and TCGA sample information (TCGA case ID, TCGA barcode, TCGA sample count, and sample total variant number). A total of 177,998 high-confidence m^6^A sites detected using base-resolution technology previously were collected and used to pinpoint the precise location of the mediated m^6^A sites within the variant-affected regions (Table S4). In addition, aspects of the post-transcriptional machinery that can be mediated by m^6^A methylation were also annotated, including RBP binding regions from POSTAR2 [88], miRNA–RNA interaction from miRanda [89] and starBase2 [90], and splicing sites from UCSC [91] annotation with GT-AG role. Furthermore, to unveil potentially related pathogenesis, any association between disease and m^6^A variants was extracted from the GWAS catalog [70] and ClinVar [71] databases.

### Database and web interface implementation

Hypertext markup language (HTML), cascading style sheets (CSS), and hypertext preprocessor (PHP) were applied to construct the m6A-TSHub web interface. All metadata were stored using MySQL tables. Besides, ECharts was exploited to present statistical diagrams, and the Jbrowse genome browser [92] was included for interactive exploration and visualization of relevant records for genome regions of interest.

## Database content and usage

### Collection of m^6^A sites from 23 normal human tissues and 25 cancer cell lines in m6A-TSDB

In m6A-TSDB, a total of 184,554 and 499,369 m^6^A-containing peaks were collected from 23 normal human tissues and 25 cancer samples, respectively. Among them, 17 out of 25 tumor samples have the m^6^A profiles of their matched primary tissues. The m^6^A-enriched peaks were called using exomePeak2 [76] with GC-correction function after mapping the processed reads to human reference genome version hg19. It is worth mentioning that, for a more complete m^6^A epitranscriptome landscape view, a total of 177,998 base-resolution m^6^A sites collected from 27 datasets using six different m^6^A profiling techniques were integrated and used to pinpoint the precise location of the mediated m^6^A sites within all tissue-specific m^6^A peaks (Table S4). In addition, all m^6^A-containing peaks were labeled with information showing whether these sites were affected by cancer somatic variants and potential involved post-transcriptional regulations. All data collected in the m6A-TSDB can be freely downloaded or shared.

### Performance evaluation and model interpretation of tissue-specific m^6^A site prediction by m6A-TSFinder

The performance of tissue-specific m^6^A site predictors was evaluated using 10-fold cross-validation and independent testing. For each distinct human tissue, we randomly selected 15% of experimentally validated m^6^A sites and used them as an independent testing dataset. For 10-fold cross-validation, the training data were randomly divided into 10 groups with the same number of positive and negative peaks. The prediction performance of each tissue-specific predictor is shown in Table 1. In general, the prediction accuracy for most tissues (20 out of the total 23 tissues) is in line with conventional approaches for m^6^A site prediction under strong supervision with base-resolution datasets, which typically reported a prediction performance between 0.8 and 0.85 in terms of the area under receiver operating characteristic (ROC) curve (AUROC) [22], [93]. The performance for kidney (AUROC = 0.718), bone marrow (AUROC = 0.757), and brainstem (AUROC = 0.789) was somewhat worse, but the reasons are not very clear. In addition, in order to find the recurring sequence patterns preferred by each tissue-specific m^6^A prediction model, we further divided the peaks into instances of length (l = 50) and extracted the consensus motifs from instances with predicted values higher than 0.5 using integrated gradient and TF-Modisco, under each tissue model, respectively. By trimming the overall letter frequencies with three gaps and two mismatches allowed, we identified one consistence motif under all tissue models (Figure S1), which was matched to the known m^6^A consensus motif DRACH. Please refer to Figure S1 for details.

**Table 1:** **Performance evaluation of tissue-specific m^6^A model**

**Tissue type**	**10-fold cross-validation**				**Independent testing**			
	**Accuracy**	**Precision**	**MCC**	**AUROC**	**Accuracy**	**Precision**	**MCC**	**AUROC**
Lung	0.764	0.835	0.536	0.843	0.775	0.761	0.55	0.853
Bladder	0.758	0.760	0.517	0.836	0.766	0.750	0.532	0.848
Colon	0.740	0.770	0.482	0.810	0.744	0.730	0.490	0.810
Lymph node	0.771	0.797	0.544	0.844	0.78	0.735	0.570	0.844
Cerebrum	0.745	0.799	0.495	0.827	0.758	0.768	0.515	0.834
Cerebellum	0.715	0.718	0.432	0.798	0.72	0.731	0.441	0.801
Hypothalamus	0.733	0.724	0.467	0.799	0.746	0.74	0.493	0.811
Brainstem	0.727	0.742	0.454	0.764	0.721	0.713	0.443	0.789
Kidney	0.685	0.694	0.369	0.755	0.647	0.628	0.297	0.718
Bone marrow	0.694	0.634	0.391	0.757	0.698	0.721	0.397	0.757
Liver	0.742	0.747	0.484	0.805	0.737	0.717	0.476	0.803
Ovary	0.730	0.710	0.464	0.814	0.726	0.722	0.453	0.812
Prostate	0.752	0.779	0.507	0.819	0.759	0.736	0.521	0.830
Soft tissue	0.766	0.855	0.544	0.855	0.771	0.775	0.543	0.858
Skin	0.750	0.850	0.511	0.835	0.773	0.753	0.547	0.857
Stomach	0.772	0.820	0.549	0.852	0.77	0.764	0.539	0.848
Corpus uterus	0.722	0.656	0.452	0.813	0.734	0.715	0.470	0.822
Adrenal gland	0.737	0.771	0.474	0.804	0.741	0.716	0.485	0.817
Heart	0.778	0.824	0.558	0.854	0.772	0.759	0.546	0.846
Rectum	0.747	0.725	0.496	0.826	0.767	0.747	0.536	0.828
Testis	0.743	0.770	0.489	0.810	0.731	0.734	0.463	0.804
Thyroid gland	0.765	0.805	0.533	0.845	0.753	0.733	0.509	0.830
Pancreas	0.761	0.770	0.523	0.838	0.751	0.739	0.502	0.834

*Note*: MCC, Matthew’s correlation coefficient; AUROC, the area under receiver operating characteristic curve.

### Performance compared with existing approaches

We further compared the performance of the proposed m6A-TSFinder with existing m^6^A predictors specifically targeted at the tissue level. Dao et al. previously developed a Support Vector Machine (SVM)-based model (iRNA-m6A) for m^6^A identification in the human brain, liver, and kidney [65]. Later, im6A-TS-CNN [64] and TS-m6A-DL [66] further improved prediction performance by applying a convolutional neural network (CNN), using the same training and testing datasets provided in Dao’s work. It is worth mentioning that the training and testing datasets used in their work contain positive and negative sequences fixed to 41-nt length with m^6^A sites or unmethylated adenosines in the center. These models learn to capture discriminative sequence patterns at positions with a fixed distance from the target adenosine. When making predictions, the well-trained models take the centered adenosine and its surrounding sequences and return the probability that the central adenosine is methylated. When only low-resolution data are available, sequence lengths vary from 100 nt to hundreds, and methylation is not fixed at the center of the sequence. Therefore, the pre-set requirements of these base-resolution models (TS-m6A-DL, im6A-TS-CNN, and iRNA-m6A) cannot be fulfilled, making it difficult to fairly evaluate their performance on low-resolution data. Furthermore, the only three tissue-specific base-resolution datasets originate from m6A-REF-seq, which can only detect m^6^A in NNACA, whereas the 23 low-resolution considered in this work contain m^6^A from broader sequence contexts. Inconsistencies between data further limit direct comparisons between models. Nevertheless, we apply m6A-TSFinder to the same training and testing datasets of the three base-resolution models to show performance and fair comparisons when base-resolution data are available. Specifically, as described in the “Data collection and processing” section, the prediction of m^6^A from fixed-length sequences centered at the target site can be considered a special case of m6A-TSFinder, in which each input sequence is treated as a single instance. As shown in Table 2, when tested on the independent dataset, m6A-TSFinder outperformed the three competing methods in two of the three tissues tested (brain and liver) and achieved the best average performance (AUROC = 0.8593). The improvement may be due to the application of the LSTM layer after the convolutional layer, which enables the model to learn the long-range dependencies between the motifs. In addition, by learning from the low-resolution datasets, we expanded the human tissues supported from 3 to 23, which could significantly facilitate future research focusing on the dynamics of m^6^A methylome across different tissues.

**Table 2:** **Performance comparison between m6A-TSFinder and competing approaches on independent dataset**

**Tissue type**	**Performance on independent dataset(AUROC)**			
	**m6A-TSFinder**	**TS-m6A-DL**	im6A**-TS-CNN**	**iRNA-**m6A
Brain	0.8132	0.8097	0.8056	0.7845
Liver	0.8850	0.8784	0.8805	0.8681
Kidney	0.8796	0.8802	0.8727	0.8565
Average	0.8593	0.8561	0.8529	0.8364

*Note*: For a fair comparison, the m6A-TSFinder was rebuilt for human brain, liver, and kidney, using the same training and testing datasets applied in the three previous studies. The 41-nt sequences were considered as one instance and fed into m6A-TSFinder.

### Assessing the impact of genetic variants on tissue-specific m^6^A sites by m6A-TSVar

The m6A-TSVar web server was designed to assess the impact of genetic variants on tissue-specific m^6^A RNA methylation using deep neural networks. The collected experimentally validated m^6^A peaks from 23 human tissues were integrated. The changes in the probability of m^6^A methylation affected by mutations were calculated, with the returned value of AL indicating how extreme the impact on m^6^A methylation was. To our best knowledge, the m6A-TSVar is the first web server for exploring m^6^A-affecting variants within a specific tissue by integrating the tissue-specific m^6^A patterns.

### Screening for cancer variants that affect m^6^A in their primary tissues in m6A-CAVar

In m6A-CAVar, the cancer somatic variants from 27 TCGA projects were extracted. Their impacts on m^6^A RNA modifications in the corresponding 23 healthy human tissues were evaluated and then systematically validated using 17 paired normal and tumor samples. A total of 587,983 cancer somatic variants were predicted to affect the m^6^A methylation status in their originating tissues (the “low-confidence level” group). Among them, the dynamic m^6^A status induced by 122,473 variants was observed on the available epitranscriptome profiles (the “medium-confidence level” group), and 1718 confirmed m^6^A-variants were known to be associated with diseases and other phenotypes from GWAS analysis (the “high-confidence level” group) (Table 3). Please refer to “Data collection and processing” section for more details related to the definition of different confidence groups.

**Table 3:** **Tissue-specific m^6^A cancer variants collected in m6A-CAVar**

**Cancer type**	**Primary tissue**	**Matched cancer cell line**	**Variant type**	**Classification**			**Total**
				**Low**	Medium	**High**	
TCGA-LUAD	Lung	A549, H1299	Gain	27,845	6526	30	34,401
			Loss	1233	1391	2	2626
TCGA-BLCA	Urinary bladder	BCa5637	Gain	25,508	3702	13	29,223
			Loss	3079	1691	6	4776
TCGA-COAD	Colon	HT29, HCT116	Gain	30,540	8391	82	39,013
			Loss	6	8284	74	8364
TCGA-DLBC	B lymphocyte cell lines	OCI-Ly1	Gain	1189	82	2	1273
			Loss	74	69	0	143
TCGA-GBM	Cerebrum	U251, GOS-3, PBT003	Gain	8509	3648	47	12,204
			Loss	1453	1181	12	2646
	Cerebellum		Gain	8319	3659	38	12,016
			Loss	1928	1271	4	3203
	Hypothalamus		Gain	6723	3414	27	10,164
			Loss	1522	1482	18	3022
	Brainstem		Gain	7559	3168	40	10,767
			Loss	1374	1451	8	2833
TCGA-KIRC	Kidney	iSLK.219	Gain	3844	227	4	4075
			Loss	54	33	0	87
TCGA-LAML	HSCs	MOLM13, THP1, NOMO-1, MONO-MAC-6, MA9.3ITD	Gain	448	274	0	722
			Loss	3	35	3	41
TCGA-LIHC	Liver	HepG2, Huh7, SMMC7721, HCCLM3	Gain	7416	2511	2	9929
			Loss	18	1765	4	1787
TCGA-OV	Ovary	PEO1	Gain	7022	531	0	7553
			Loss	1350	1090	6	2446
TCGA-PRAD	Prostate gland	Cd-RWPE-1	Gain	3825	636	6	4467
			Loss	550	288	2	840
TCGA-SARC	Soft tissues	U20S	Gain	3592	1324	4	4920
			Loss	373	28	0	401
TCGA-SKCM	Skin	Mel624	Gain	79,470	17,177	118	96,765
			Loss	6472	1559	2	8033
TCGA-STAD	Stomach	BGC823	Gain	35,438	2202	34	37,674
			Loss	1103	3313	27	4443
TCGA-UCEC	Corpus uteri	HEC-1-A	Gain	80,712	38,242	266	119,220
			Loss	7813	1828	22	9663
TCGA-LUSC	Lung	–	Gain	31,106	–	118	31,224
			Loss	2328	–	2	2330
TCGA-MESO	Lung	–	Gain	595	–	4	599
			Loss	57	–	0	57
	Heart	–	Gain	674	–	5	679
			Loss	102	–	0	102
TCGA-LGG	Cerebrum	–	Gain	6714	–	92	6806
			Loss	1423	–	19	1442
	Cerebellum	–	Gain	6601	–	109	6710
			Loss	1745	–	16	1761
	Hypothalamus	–	Gain	5010	–	77	5087
			Loss	1698	–	11	1709
	Brainstem	–	Gain	5740	–	114	5854
			Loss	1528	–	13	1541
TCGA-KICH	Kidney	–	Gain	484	–	9	493
			Loss	9	–	0	9
TCGA-KIRP	Kidney	–	Gain	4028	–	17	4045
			Loss	118	–	0	118
TCGA-CHOL	Liver	–	Gain	728	–	2	730
			Loss	166	–	2	168
TCGA-ACC	Adrenal gland	–	Gain	2285	–	21	2306
			Loss	385	–	3	388
TCGA-PCPG	Adrenal gland	–	Gain	345	–	1	346
			Loss	57	–	0	57
TCGA-READ	Rectum	–	Gain	12,433	–	100	12,533
			Loss	1098	–	4	1102
TCGA-THYM	Heart	–	Gain	520	–	7	527
			Loss	80	–	2	82
TCGA-TGCT	Testis	–	Gain	405	–	3	408
			Loss	109	–	0	109
TCGA-THCA	Thyroid gland	–	Gain	992	–	6	998
			Loss	156	–	0	156
TCGA-PAAD	Pancreas	–	Gain	6473	–	55	6528
			Loss	1236	–	3	1239
Total	–	–	–	463,792	122,473	1718	587,983

*Note*: TCGA-LUAD, The Cancer Genome Atlas Lung Adenocarcinoma; TCGA-BLCA, The Cancer Genome Atlas Bladder Urothelial Carcinoma; TCGA-COAD, The Cancer Genome Atlas Colon Adenocarcinoma; TCGA-DLBC, The Cancer Genome Atlas Lymphoid Neoplasm Diffuse Large B-cell Lymphoma; TCGA-GBM, The Cancer Genome Atlas Glioblastoma Multiforme; TCGA-KIRC, The Cancer Genome Atlas Kidney Renal Clear Cell Carcinoma; TCGA-LAML, The Cancer Genome Atlas Acute Myeloid Leukemia; TCGA-LIHC, The Cancer Genome Atlas Liver Hepatocellular Carcinoma; TCGA-OV, The Cancer Genome Atlas Ovarian Serous Cystadenocarcinoma; TCGA-PRAD, The Cancer Genome Atlas Prostate Adenocarcinoma; TCGA-SARC, The Cancer Genome Atlas Sarcoma; TCGA-SKCM, The Cancer Genome Atlas Skin Cutaneous Melanoma; TCGA-STAD, The Cancer Genome Atlas Stomach Adenocarcinoma; TCGA-UCEC, The Cancer Genome Atlas Uterine Corpus Endometrial Carcinoma; TCGA-LUSC, The Cancer Genome Atlas Lung Squamous Cell Carcinoma; TCGA-MESO, The Cancer Genome Atlas Mesothelioma; TCGA-LGG, The Cancer Genome Atlas Brain Lower Grade Glioma; TCGA-KICH, The Cancer Genome Atlas Kidney Chromophobe; TCGA-KIRP, The Cancer Genome Atlas Kidney Renal Papillary Cell Carcinoma; TCGA-CHOL, The Cancer Genome Atlas Cholangiocarcinoma; TCGA-ACC, The Cancer Genome Atlas Adrenocortical Carcinoma; TCGA-PCPG, The Cancer Genome Atlas Pheochromocytoma And Paraganglioma; TCGA-READ, The Cancer Genome Atlas Rectum Adenocarcinoma; TCGA-THYM, The Cancer Genome Atlas Thymoma; TCGA-TGCT, The Cancer Genome Atlas Testicular Germ Cell Tumors; TCGA-THCA, The Cancer Genome Atlas Thyroid carcinoma; TCGA-PAAD, The Cancer Genome Atlas Pancreatic Adenocarcinoma; HSC, hematopoietic stem cell.

### Deciphering the tissue specificity of cancer m^6^A variants

**Figure 4:**
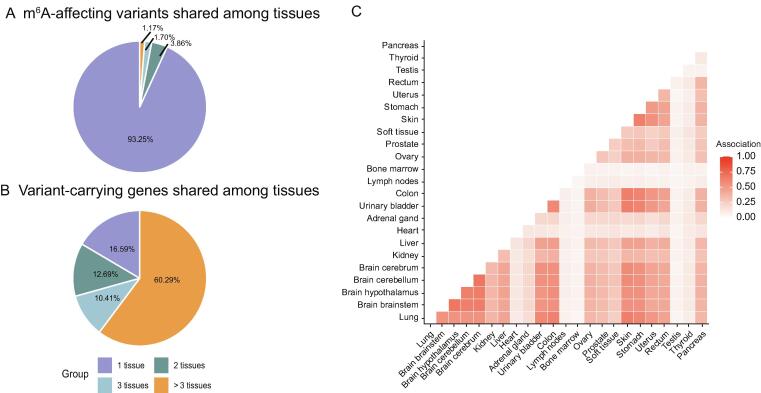
**Tissuespecificity of cancer m^6^A variants** **A.** The proportion of m^6^A variants that are shared among different tissues. Most m^6^A-associated variants (93.25%) were identified in only 1 tissue, with 3.86%, 1.70%, and 1.17% identified in 2, 3, and more than 3 tissues, respectively. **B.** The proportion of m^6^A variant-carrying genes shared among tissues. The consistency is much higher at the gene level. Most m^6^A variants-carrying genes are shared among multiple tissues, with only 16.59% associated to one tissue type. **C.** The pairwise association of tissues in terms of proportion of shared m^6^A variant-carrying genes. Most tissues are significantly correlated. The exceptions are heart, adrenal gland, lymph nodes, bone marrow, testis, and thyroid.

Of interest is whether m^6^A variants function in different cancer-originating tissues. For this purpose, we calculated the proportion of m^6^A variants that function in different numbers of tissues, and the results suggested that most m^6^A-associated cancer variants are tissue- and cancer-specific (93.25%), whereas only around 1.17% are functional in the originating tissues of more than three types of cancers (Figure 4A). The consistency is much higher at the gene level. Only around 16.59% of m^6^A variant-carrying genes are associated with a single tissue. More than 60.29% were shared in more than three tissue types (Figure 4B), suggesting some common epitranscriptome layer circuitry at the gene level in different cancers. We further examined the proportion of shared m^6^A variant-carrying genes between two different tissues. As shown in Figure 4C, most tissues, *e.g.*, skin and stomach, strongly correlated with each other. However, tissues like the heart, testis, and thyroid showed a rather weak association with other tissues, which may suggest more tissue-specific epitranscriptome circuitry for cancers originating in those tissues.

We finally identified the m^6^A variant-carrying genes that are associated with the most TCGA cancer types. Only experimentally validated m^6^A variants (medium confidence level and above) were considered here for a more reliable analysis. Top of the list was *CENPF,* in which variants may change its m^6^A methylation status in the primary tissue of 15 cancer types, followed by *DST*, *MKI67*, and *PLEC*, which were all related to 14 cancer types (detailed in Table S5). Among them, the roles in epitranscriptome regulation of *CENPF*, *MKI67*, and *PLEC* have been indicated previously in glioblastoma [94], breast cancer [95], and pancreatic cancer [96], respectively.

### Enhanced web interface and application

**Figure 5:**
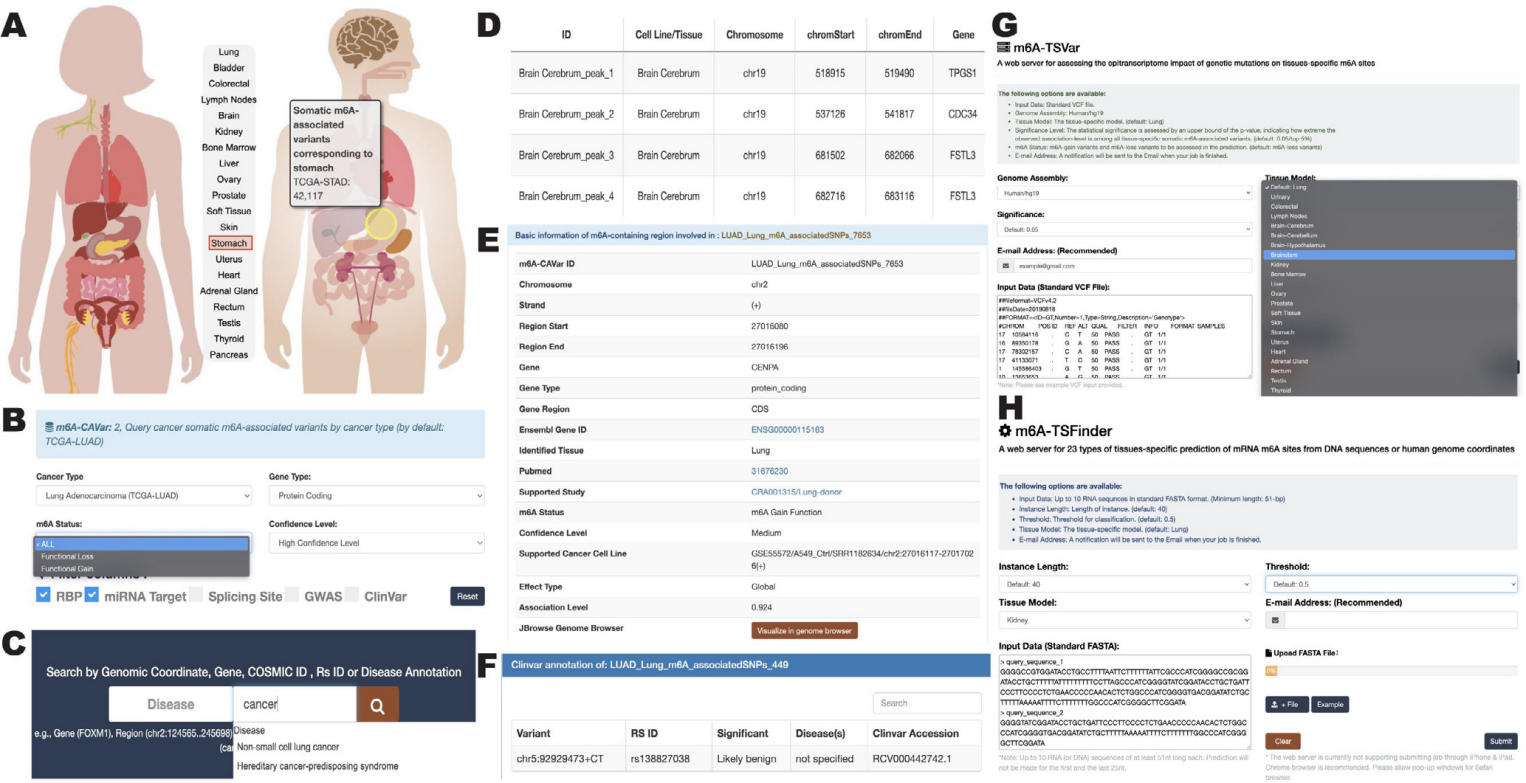
**Enhanced web interface** **A.** A human body diagram is available for querying cancer somatic m^6^A-associated variants in their originating tissues. **B.** Users can query the associated variants by cancer type. **C.** Users can also query the variant-associated disease, region, gene symbol, COSMIC, and Rs ID, and further filter the returned results. **D.** Details of tissue-specific m^6^A peaks collected in m6A-TSDB. **E.** Details of cancer-related m^6^A-associated variants. **F.** Details of disease annotation involved. **G.** The online tools provided for analysis of user-uploaded files, including assessing m^6^A-associated variants in tissues (m6A-TSVar). **H.** The online tool for identifying tissue-specific m^6^A sites (m6A-TSFinder).

The m6A-TSHub features a user-friendly web interface with multiple useful functions, including databases and online servers, which enable users to fast query databases, upload their own custom jobs, and download all m^6^A-related information at the tissue level. The collected functional m^6^A-affecting variants can be queried by a human body diagram according to their primary tissues (Figure 5A), as well as by different cancer types along with further filters (*e.g.*, gene type, m^6^A status, confidence level, and disease association; Figure 5B). The query function also returns several categories of useful information, including TCGA project names [77], tumor-growth tissues, genes, chromosome regions, COSMIC ID [97], and disease phenotypes (Figure 5C). The details of tissue-specific m^6^A peaks collected in m6A-TSDB (Figure 5D) and cancer m^6^A-associated variants in m6A-CAVar (Figure 5E) can be viewed by clicking the site or variant ID, along with annotated disease-association regulations (Figure 5F). Furthermore, online servers allow for the identification of m^6^A sites and m^6^A-associated variants within user-defined regions, with 23 types of human tissues to be selected (Figure 5G and H). A genome browser is available for interactive exploration of the genome regions of interest, including the human gene annotation track, 23 normal tissue tracks, 25 cancer cell line tracks, single-base m^6^A epitranscriptome landscape track, and post-transcriptional regulation tracks. All metadata provided in the m6A-TSHub can be freely downloaded, along with server scripts provided to run the prediction tools locally (required language: R and Python). Users can refer to the ‘help’ and ‘download’ page for more detailed guidance and instructions.

### Case study 1: *PIK3CA* variant in colon cancer

Previous studies have reported that m^6^A RNA modification plays an important role in colon cancer [61], [98], [99], [100]. The Cancer Genome Atlas Colon Adenocarcinoma (TCGA-COAD) project [77] presented a large number of somatic variants identified from various colon adenocarcinoma samples. However, it is still unclear which single genetic variant may lead to m^6^A dysregulation. In m6A-CAVar, a somatic variant at Chr3:178952085 (A > T) on *PIK3CA* identified from TCGA-COAD project (TCGA barcode: TCGA-AA3821-01A-01W-0995-10) was predicted to erase the m^6^A methylation of a region (Chr3:178951888-178952363, positive strand). The m^6^A methylation was observed in healthy human colon, but disappeared in the colon adenocarcinoma cancer cell line HCT116 [101]. This somatic variant is also recorded in the COSMIC database from colon tumor samples under the legacy identifier of COSM776, and reported to be associated with 27 submitted interpretations and evidence in the ClinVar database [71], including PIK3CA-related overgrowth spectrum (ClinVar accession: RCV000201235.1), breast adenocarcinoma (ClinVar accession: RCV000014629.5), and pancreatic adenocarcinoma (ClinVar accession: RCV000417557.1). Taken together, these observations strongly support the functional importance of this variant. Additionally, the m^6^A-associated variant falls within the binding regions of two RBPs (TARDBP and NUDT21), whose interaction may be regulated by the loss of m^6^A methylation in the cancer condition, providing some putative downstream regulatory consequences of the variant.

### Case study 2: *PLEC* variant in glioblastoma

Glioblastoma (GBM) is the most aggressive type of brain tumor and is associated with rising mortality. The roles of m^6^A regulators in this disease have been previously indicated [102], [103], [104], [105]. A somatic cancer variant on *PLEC* was identified from the The Cancer Genome Atlas Glioblastoma Multiforme (TCGA-GBM) project (TCGA barcode: TCGA-06-5416-01A-01D-1486-08) at Chr8:144991388 (C > T). This cancer variant was predicted to lead to a gain of an m^6^A site on a previously unmethylated region in a healthy human cerebrum. Indeed, an m^6^A site was detected in this region from malignant GBM tumor cell line U-251. This mutation has a record in ClinVar database (ClinVar accession: RCV000177727.1). Screening for potential post-transcriptional regulations revealed that the cancer variant falls within the target binding regions of six RBPs, including the m^6^A reader YTHDF1, which is known to bind m^6^A-containing RNAs and promote cancer stem cell properties of GBM cells [106]. It should be of immediate interest to ask whether the methylation of *PLEC* regulates its interaction with YTHDF1 and other RBPs, and what the functional consequences are.

### Case study 3: *EGFR* variant in lung cancer

**Figure 6:**
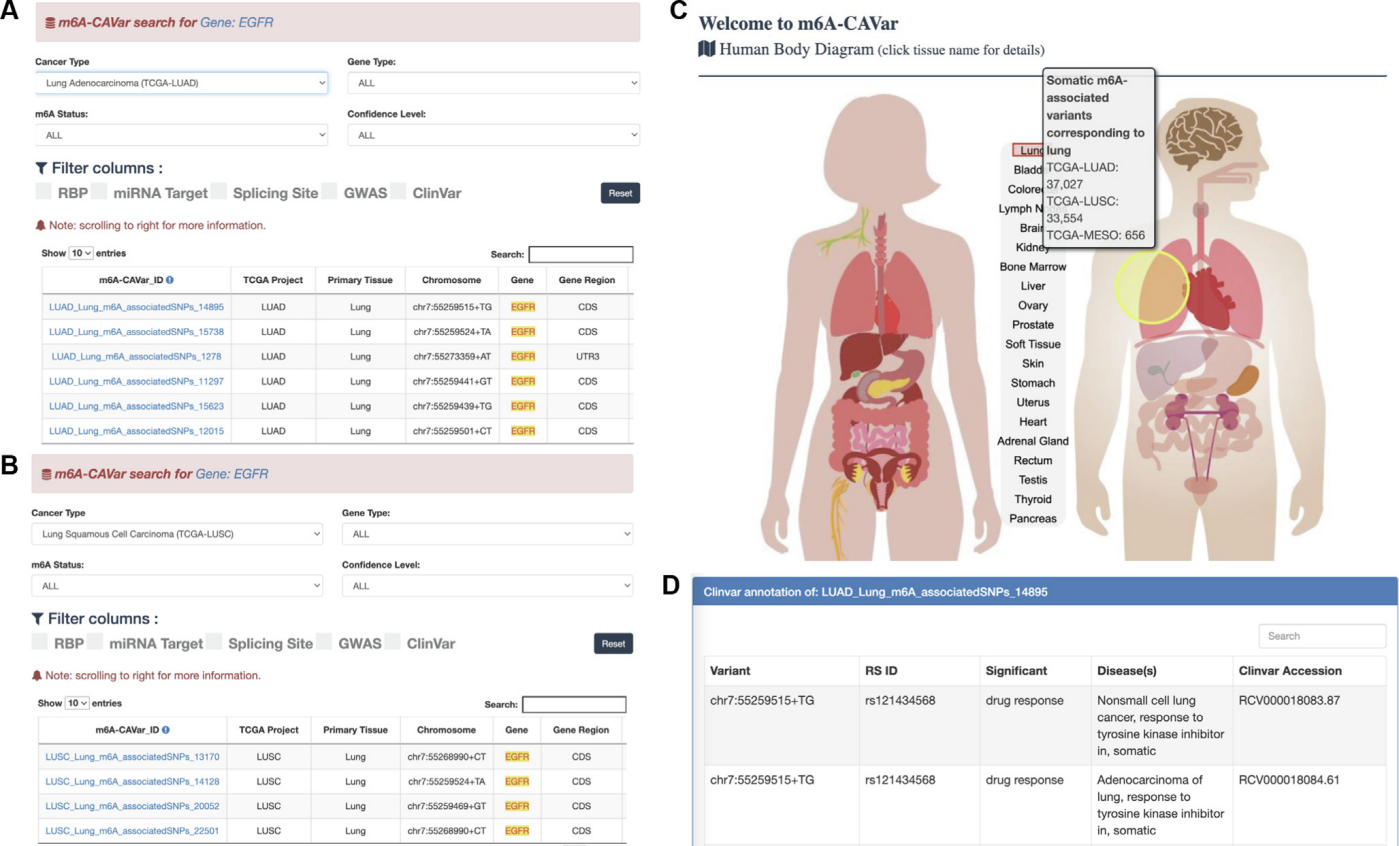
**Case study onthe*EGFR*gene** **A.** Searching for the gene *‘EGFR’* in m6A-CAVar database returns a total of 10 m^6^A variants identified in two lung cancer types. The details of which can be viewed by clicking the m6A-CAVar ID. **B.** Users can further filter the search results in specific cancer types. **C.** A human body map provided on the front page of m6A-CAVar website. It enables quick positioning of cancer m^6^A-associated variants functioning at a specific tissue. **D.** The disease and phenotype associations of recorded m^6^A variant.

The associations between m^6^A RNA modifications and human lung cancers have been well studied. The m^6^A eraser FTO may be a prognostic factor in The Cancer Genome Atlas Lung Squamous Cell Carcinoma (TCGA-LUSC) [107], and the m^6^A writer METTL3 regulates *EGFR* expression to promote cell invasion of human lung cancer cells [82]. The m6A-CAVar database can be used to explore the role of m^6^A variants of *EGFR* in lung cancers. We first search by gene name ‘*EGFR*’ on the front page of the m6A-CAVar database, then filter the results and keep only records related to lung tissue, which retains a total of 10 cancer m^6^A-associated variants from two lung cancer types (Figure 6A and B). Alternatively, the users can query all recorded m^6^A-associated variants that function in lung tissue by simply clicking the relevant part from the human body diagram (Figure 6C). More details can be accessed by clicking the variant ID. For example, if we check further details of an m^6^A-gain variant from the TCGA-LUAD project at Chr7:55259515 (T > G), we can see that this variant is recorded in the ClinVar database and is relevant to eight disease conditions, including lung cancers (Figure 6D), which may suggest potential cancer pathogenesis originates in the epitranscriptome layer.

## Discussion and perspectives

The context-specific expression and functions of m^6^A regulations have been repeatedly reported in existing studies [59], [60], [61], [62], [63], suggesting the involvement of the tissue-specific m^6^A methylome in essential biological processes and multiple disease mechanisms. Besides, the associations between RNA methylation levels and the activities of RNA methylation regulators were clearly unveiled, reporting that there exist some condition-specific RNA co-methylation patterns (a group of RNA m^6^A methylation sites whose methylation levels go up and down together) [108], [109], [110]. These co-methylation patterns are enriched by the substrate targets of m^6^A regulators and thus are probably regulated by specific m^6^A methyltransferase or demethylase.

Here, we present m6A-TSHub, a comprehensive online platform for unveiling the context-specific m^6^A methylation and m^6^A-affecting mutations in 23 human tissues and 25 tumor conditions. In m6A-TSHub, a total of 184,554 and 499,369 m^6^A sites derived from 23 normal human tissues and 25 matched tumor samples were collected (m6A-TSDB), from which some potential patterns for the tissue-specific m^6^A modification sites were revealed (*e.g.*, heart-enriched genes *RYR2* and *PXDNL*; Figure S2). Based on these collected data, 23 distinct m^6^A prediction models were built at the tissue level using deep neural networks (m6A-TSFinder). In addition, to elucidate the genetic factor of epitranscriptome dysregulation, m6A-CAVar identified a total of 587,983 cancer somatic mutations that may alter the m^6^A status in corresponding cancer originating tissues and annotated them with various functional annotations, including features relating to post-transcriptional regulations (RBP binding regions, miRNA targets, and splicing sites), disease and phenotype associations, as well as other useful genomic information (transcript structure, phastCons, and deleterious level) to provide a more comprehensive overview. We also provide a web server m6A-TSVar for assessing the effect of genetic variants on m^6^A methylation in a specific tissue.

Although most of the existing approaches for RNA modification site prediction ignore the tissue-specific signatures of m^6^A methylation, by taking advantage of existing tissue-specific epitranscriptome data, our method can predict the m^6^A methylation within a specific tissue. Compared with existing approaches for tissue-specific m^6^A methylation site prediction [64], [65], [66], our approach m6A-TSFinder achieved a higher prediction performance (Table 2) and hugely expanded the number of supported tissue types from 3 to 23 (Table 1).

Compared with existing approaches for decoding the epitranscriptome impact of genetic variants, m6A-CAVar has the following two major advantages. First, m6A-CAVar relies on a finer prediction model (m6A-TSFinder) that appreciates the specific pattern of RNA methylomes across different tissues. By directly learning from the epitranscriptome profiles in 23 healthy human tissues, m6A-CAVar is able to evaluate the tissue-specific impact of cancer somatic variants on m^6^A modification in their originating tissue, providing a more detailed picture of the genome–epitranscriptome association. This improves on existing approaches that ignore the distinct signatures of RNA methylation across different tissues and thus fail to address tissue-specific effects. Second, the predicted m^6^A dynamics in m6A-CAVar were systematically validated using available epitranscriptome datasets from the matched healthy and cancerous samples, providing another layer of quality assurance from real omic datasets. In contrast, existing approaches use those datasets only to provide the m^6^A site information without searching for potential evidence of m^6^A status switching.

To date, epitranscriptome data are still rather scarce. Due to the limited availability of datasets, matched healthy tissue and cancer m^6^A profiling samples are only available for 14 out of the total 27 cancer types, prohibiting a more thorough validation of the predicted results. Furthermore, a substantial discrepancy has been observed among different RNA modification profiling approaches due to technical biases [111], [112], [113], [114], which can produce additional inaccuracy. Currently, context-specific epitranscriptome prediction is only possible for a small number of conditions (cell line, tissue type, and treatment) with data [64], [65], [66]. However, the m6A-TSHub framework will be further expanded when epitranscriptome datasets are more abundantly available for more comprehensive and less biased screening of context-specific m^6^A-variants, along with linking the tissue-specific epitranscriptome patterns with other important cancer-associated factors such as human aging [67], [115]. Besides, the current version of m6A-TSHub was built on human genome assemble hg19. A LiftOver file from hg19 to hg38 was provided on the ‘download’ page, and the next version of the database will be updated based on the latest genome assembly. Particularly promising is the recent development in Nanopore direct RNA sequencing technology that enables simultaneous identification of multiple RNA modifications with simplified sample preparation procedures [116], [117], [118], [119], [120], [121], [122], [123], [124].

## Data availability

The data underlying this article are available at www.xjtlu.edu.cn/biologicalsciences/m6ats. The online versions of the m6A-TSFinder and m6A-TSVar web server are available at www.xjtlu.edu.cn/biologicalsciences/m6ats by clicking the ‘tool’ section. The local version and project codes can be accessed on the ‘download’ page.

## Competing interests

The authors declare that they have no competing interests.

## CRediT authorship contribution statement

**Bowen Song:** Methodology, Data curation, Software, Visualization, Writing – original draft. **Daiyun Huang:** Software, Supervision. **Yuxin Zhang:** Visualization. **Zhen Wei:** Resources. **Jionglong Su:** Visualization. **João Pedro de Magalhães:** Visualization. **Daniel J. Rigden:** Writing – review & editing. **Jia Meng:** Conceptualization, Supervision, Writing – review & editing, Funding acquisition. **Kunqi Chen:** Conceptualization, Resources, Writing – review & editing, Funding acquisition. All authors have read and approved the final manuscript.

## Supplementary material

Supplementary Figure S1 **Motif captured under each tissue-specific m6A prediction model** The consensus motifs from instances with higher than average weights were extracted using TF-MoDISco, under each tissue model, respectively. To sum up, we identified one consistence motif GGACU under all tissue models, which was matched to the known m6A consensus motif DRACH.

Supplementary Figure S2 **m6A patterns captured under specific human gene A.** human gene *RYR2* encodes a ryanodine receptor found in cardiac muscle sarcoplasmic reticulum, this gene was biased expressed in heart and brain. We found three m6A sites located on gene *RYR2* from heart samples, compared with two m6A sites from brain, one from liver, one from ovary, and one from uterus, respectively. **B.** for human gene *PXDNL* (biased expression in heart), we observed only one tissue-specific m6A sites from heart sample. **C.** and **D.** human gene *HMGCS2* and *C6* were both reported to be biased expressed in liver. We found one m6A peak located on gene *HMGCS2* and gene *C6*, respectively, identified from human liver sample.
