# Supplementary material for: m6A-TSHub: Unveiling the Context-specific m6A Methylation and m6A-affecting Mutations in 23 Human Tissues
Source: Genomics Proteomics Bioinformatics. 2022 Sep 9;21(4):678–94. doi: 10.1016/j.gpb.2022.09.001 (PMC10787194; doi:10.1016/j.gpb.2022.09.001)
Supplement: Supplementary Figure S1 — Motif captured under each tissue-specific m6A prediction model The consensus motifs from instances with higher than average weights were extracted using TF-MoDISco, under each tissue model, respectively. To sum up, we identified one consistence motif GGACU under all tissue models, which was matched to the known m6A consensus motif DRACH. [file mmc1.pptx]

## Slide 1
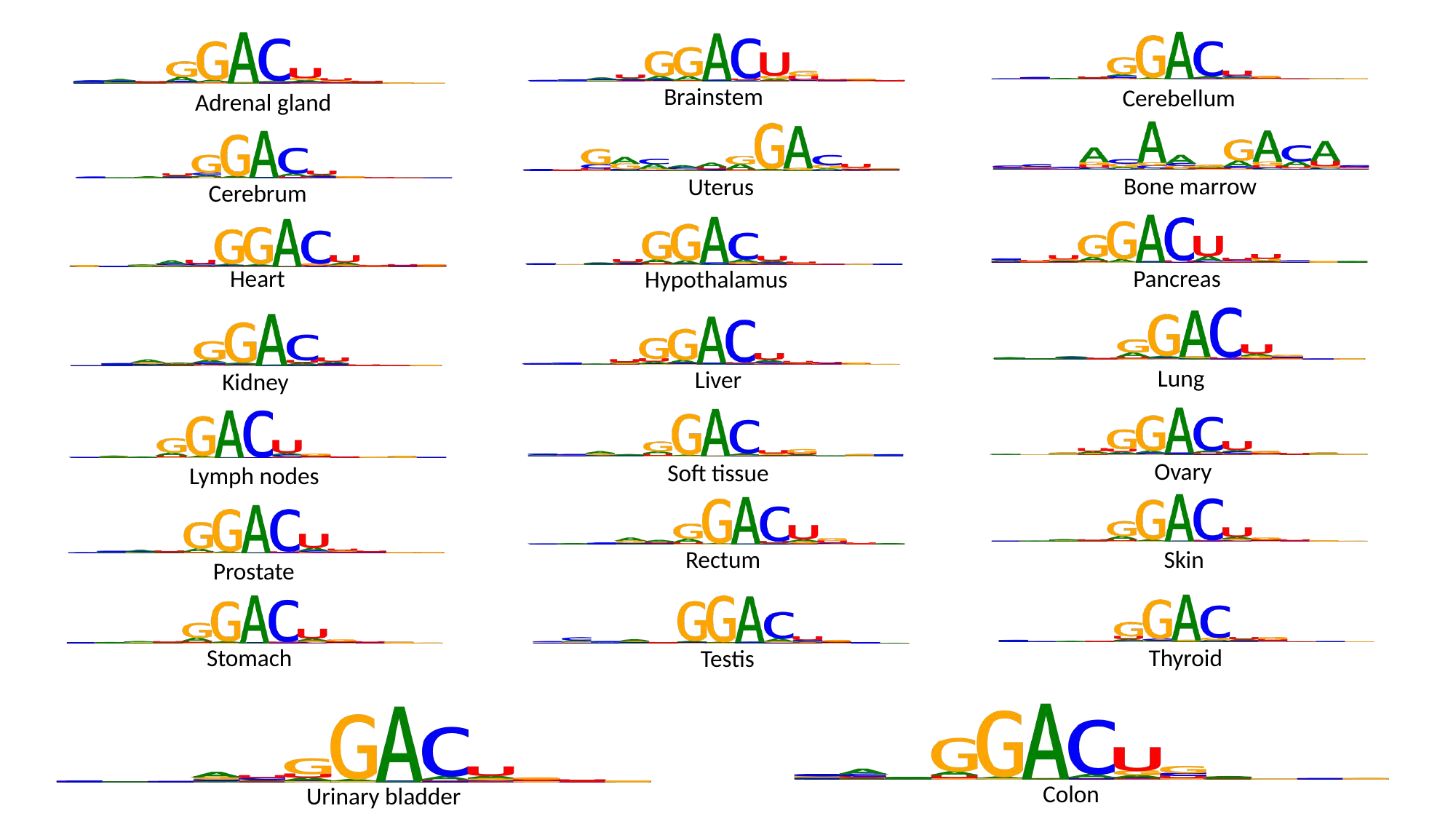

Brainstem
Cerebellum
Adrenal gland
Bone marrow
Uterus
Cerebrum
Heart
Pancreas
Hypothalamus
Lung
Liver
Kidney
Ovary
Soft tissue
Lymph nodes
Rectum
Skin
Prostate
Stomach
Thyroid
Testis
Colon
Urinary bladder
