# Supplementary figures and images for: m6A-TSHub: Unveiling the Context-specific m6A Methylation and m6A-affecting Mutations in 23 Human Tissues
Source: Genomics Proteomics Bioinformatics. 2022 Sep 9;21(4):678–94. doi: 10.1016/j.gpb.2022.09.001 (PMC10787194; doi:10.1016/j.gpb.2022.09.001)

## Slide 1
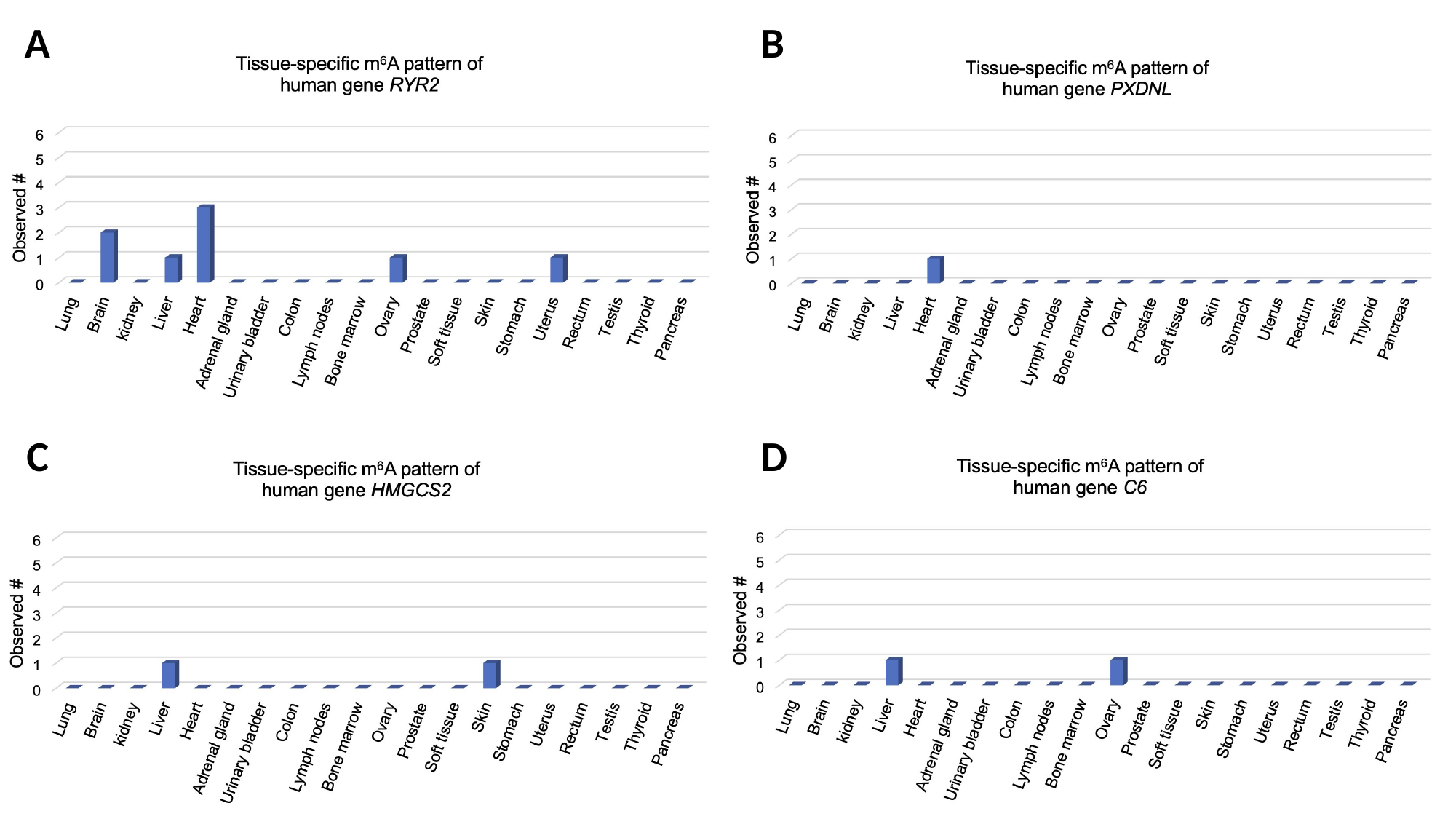

A
B
C
D

Supplement: Supplementary Figure S2 — m6A patterns captured under specific human gene A. human gene RYR2 encodes a ryanodine receptor found in cardiac muscle sarcoplasmic reticulum, this gene was biased expressed in heart and brain. We found three m6A sites located on gene RYR2 from heart samples, compared with two m6A sites from brain, one from liver, one from ovary, and one from uterus, respectively. B. for human gene PXDNL (biased expression in heart), we observed only one tissue-specific m6A sites from heart sample. C. and D. human gene HMGCS2 and C6 were both reported to be biased expressed in liver. We found one m6A peak located on gene HMGCS2 and gene C6, respectively, identified from human liver sample. [file mmc2.pptx]
