# Supplementary material for: m6A-TSHub: Unveiling the Context-specific m6A Methylation and m6A-affecting Mutations in 23 Human Tissues
Source: Genomics Proteomics Bioinformatics. 2022 Sep 9;21(4):678–94. doi: 10.1016/j.gpb.2022.09.001 (PMC10787194; doi:10.1016/j.gpb.2022.09.001)
Supplement: Supplementary Table S1 [file mmc3.docx]

**Table S1 Distinct features of m^6^A-TSHub compared with existing resources**

| **Feature & function** | **m^6^A-TSHub** | **RMDisease** | **RMVar** | **m^7^GHub** | **RMBase v2.0** | **m^6^A-Atlas** |
| --- | --- | --- | --- | --- | --- | --- |
| m^6^A prediction model | 23 individual context-specific model | One single model for all conditions | One single model for all conditions | One single model for all conditions | - | - |
| Mutations  analysis | TCGA cancer mutations analyzed in corresponding tumor-originating tissues | Mutations matched to all conditions | Mutations matched to all conditions | Mutations matched to all conditions | Only mutations destroying m6A motif reported | Only mutations destroying m6A motif reported |
| Validation of predicted m^6^A dynamics | Validation by omics datasets in cancer conditions | - | - | - | - | - |
| Context-specific collection of m^6^A sites | Explicitly and more complete  (m^6^A-TSDB) | - | - | m^7^G sites with no tissue-specific collection | Inexplicitly, limited volume | Inexplicitly, limited volume |
| Web server function | m^6^A-TSFinder  & m^6^A-TSVar | - | - | Not supported for context-specific analysis | - | Not supported for m^6^A identification and mutation analysis |
| Query function by COSIMC ID | Yes | - | - | - | - | - |
